# Preventing Chronic Illness in Young Veterans by Promoting Healthful Behaviors

**Published:** 2011-12-15

**Authors:** Rachel Widome, Alyson J. Littman, Melissa N. Laska, Steven S. Fu

**Affiliations:** Center for Chronic Disease Outcomes Research (CCDOR), Minneapolis VA Medical Center; VA Seattle Epidemiologic Research and Information Center (ERIC), VA Puget Sound Medical Center, the Department of Epidemiology, School of Public Health, University of Washington, Seattle, Washington; University of Minnesota School of Public Health, Minneapolis, Minnesota; CCDOR, Minneapolis VA Medical Center and Department of Medicine, University of Minnesota, Minneapolis, Minnesota

Since October 2001, more than 2 million Americans have served in the US wars in Afghanistan and Iraq, and nearly half of these veterans have been deployed more than once (1). Most are adults younger than age 35 when they return home from service. Upon return, veterans can choose to remain active, be inactive while serving in a component such as the Reserves or National Guard, or be separated from service. In the general population, the transition from adolescence to young adulthood is a time of increased risk for behavioral chronic disease risk factors such as excess weight gain and tobacco use escalation. However, few studies have examined the health behaviors of young veterans, and, perhaps as a result, few programs, interventions, and policies are designed to promote healthful behaviors for recently returned veterans. There are a variety of reasons as to why veterans are at increased risk for chronic disease risk behavior. We will also highlight opportunities to develop innovative strategies to promote healthful behaviors among veterans of the wars in Iraq and Afghanistan.

## Gaps in Our Understanding of Tobacco-Use and Weight-Related Behaviors Among Young Veterans

Three modifiable behaviors — tobacco use, physical inactivity, and poor diet — caused one-third of all deaths in the United States in 2000 (2,3). Promoting tobacco-free lifestyles and healthful weight-related behaviors is important in the veteran population, which appears to be at increased risk for some of these behaviors (4,5).

The prevalence of tobacco use among veterans of the wars in Iraq and Afghanistan is high. In 2008, nearly one-third of active duty military personnel reported smoking in the past month, and 14% reported smokeless tobacco use (6); meanwhile, just under 20% of the adult US population were reported to be current smokers (7), and approximately 3% of the US population older than aged 12 years reportedly used smokeless tobacco (8). Although military service has long been associated with tobacco use, the prevalence of tobacco use among Iraq and Afghanistan war veterans appears to be exceedingly high; they are 50% more likely to use tobacco than their military peers who did not deploy (4). Some never smokers and most former smokers who deploy to Iraq and Afghanistan initiate or resume smoking (5). A complex array of factors that include sociocultural background, personality traits that may be more common to people who join the military, combat exposure, military culture, reintegration challenges, military career path, alcohol abuse, and emotional or mental health issues likely underlies this high prevalence of tobacco use, which has also been observed in prior conflicts (4,5).

For the general population, emerging adulthood is a time of increased risk for excess weight gain (9), and the prevalence of obesity among young adults aged 20 to 39 years is high; more than one-fourth of young adults are obese (body mass index [BMI] >30 kg/m^2^) (10). Data on obesity rates in Afghanistan and Iraq war veterans are limited. Iraq and Afghanistan war veterans in a US Department of Veterans Affairs (VA) sample were more likely to be overweight but less likely to be obese compared with national same-age samples (11). In a large military cohort study, nearly half of participants experienced "extreme weight gain" (≥10% of their weight) from the first wave of data collection (2001-2003) through the second wave (2004-2006), a period of time when much of the sample deployed to Iraq or Afghanistan (12). Furthermore, Iraq and Afghanistan war veterans who are overweight or obese are at increased risk for hypertension (13). Compared with their nonveteran peers, young adult veterans may be more affected by stress, depression, substance and alcohol abuse, and sleep loss, all of which have been linked to weight-related behaviors and obesity (14).

Although it is easy to focus on risk behaviors, there are also potentially strong protective factors relating to tobacco use and weight gain that may be leveraged to reduce chronic disease risk. For instance, military culture highly values physical fitness. Evidence exists that the veterans of the Iraq and Afghanistan wars are more likely to engage in strength training compared with their nonveteran peers (15). Additionally, the military breeds a strong sense of camaraderie and community, which can counteract stress and potentially assist with making a behavior change such as quitting tobacco use or changing one's diet. A structural asset for this population is the VA health care system. All veterans of the Iraq and Afghanistan wars are eligible for at least 5 years of care through the VA after they separate from military service. Although the VA, like most health care systems, has had more focus and expertise in chronic disease treatment, this infrastructure could be channeled for primary prevention.

## A Unique Window of Opportunity

Both tobacco use and weight-related behavior patterns appear to be established during young adulthood and persist throughout life. Although most smokers had their first cigarette while they were adolescents, most young smokers have not established their smoking pattern before they reach age 18. Young adulthood appears to be the period when smoking patterns "lock in," as few people initiate or quit smoking in the decade following young adulthood (in this particular study, ages 28-40 y) (16). Weight-related behaviors also appear to remain consistent after young adulthood (14). This finding suggests that the period after deployment may be an effective time to attempt to set a beneficial health behavior trajectory.

Another reason to intervene during young adulthood is that the process of developing chronic disease begins often decades before symptoms emerge. For instance, most of the deaths associated with smoking can be avoided by quitting smoking by age 35 (17). Consequently, eliminating tobacco use at a young age would have an immense health impact for the population of young adult Afghanistan and Iraq war veterans who smoke.

## Proactively Promoting Healthful Behaviors Among Young Veterans

What is needed to develop interventions, opportunities, policies, and systems that promote healthful lifestyles for returning veterans? First, we need more data that can describe the magnitude and correlates of the problem of risk behaviors in the population of young veterans. Our data-gathering processes must be updated to be consistent with 21st-century young adult culture. For instance, researchers and research institutions should be open to recruiting participants for studies by using social networking websites, surveying online, and formulating questions that are relevant to young adults. The data should tell us both who is at risk and how personal, cognitive, and environmental factors contribute to the establishment of behaviors and enable or impede behavior change. In the absence of knowledge about these issues in this new cohort of veterans, promoting more healthful lifestyles and providing preventive health services aimed at reducing health risk behaviors among Iraq and Afghanistan war veterans is an insurmountable challenge.

The next step is to develop innovative interventions and craft policies that steer young veterans toward healthful behaviors long before signs and symptoms of chronic disease appear. Policies such as improving the nutrition of food served at National Guard drill weekends may contribute to positive dietary change. Other interventions could include developing opportunities for physical activity for veterans, such as fitness classes held at VA clinics. Ensuring that all Iraq and Afghanistan war veterans have access to free evidence-based tobacco use cessation aids could also reduce barriers to cessation.

To reach young veterans, we need to establish partnerships that cut across traditional institutional domains, for example. Although the VA is the largest health care provider to veterans, many returning veterans are reluctant to use VA services. Since the wars began, only 51% of eligible Iraq and Afghanistan war veterans have sought care through the VA (1). This is in part because of unfamiliarity with the system, distance from the VA medical centers, misconceptions about the quality of VA care, or reluctance to visit a large VA hospital for routine care. One pressing issue in providing any kind of services to veterans, especially those who have separated recently, is that the Military Health System and VA are not well-integrated, which makes continuity of any type of service more challenging. The Post-9/11 Veterans Educational Assistance Act of 2008 has enabled hundreds of thousands of Iraq and Afghanistan war veterans to attend college (18-19). Some natural partnerships could emerge between universities and organizations devoted to promoting veteran health.

Adoption of healthful lifestyles has the benefits of improved health, reduced disease, and an enhanced quality of life for years to come. As young Iraq and Afghanistan war veterans return, many will interact with organizations that have a stake in their well-being, which represents an opportunity to emphasize ways to prevent chronic illness in this population.
